# Evaluation of the effectiveness of BG-Sentinel and CDC light traps in assessing the abundance, richness, and community composition of mosquitoes in rural and natural areas

**DOI:** 10.1186/s13071-022-05172-3

**Published:** 2022-02-08

**Authors:** André B. B. Wilke, Chalmers Vasquez, Augusto Carvajal, Maday Moreno, William D. Petrie, John C. Beier

**Affiliations:** 1 Department of Public Health Sciences, Miller School of Medicine, University of Miami, 1120 Northwest 14th Street, Miami, FL 33136 USA; 2 Miami-Dade County Mosquito Control Division, Miami, FL USA

**Keywords:** Mosquitoes, Mosquito surveillance, Vector-borne diseases, Arboviruses, Malaria

## Abstract

**Background:**

Vector-borne diseases are a major burden to public health. Controlling mosquitoes is considered the most effective way to prevent vector-borne disease transmission. Mosquito surveillance is a core component of integrated vector management, as surveillance programs are often the cornerstone for the development of mosquito control operations. Two traps are the most commonly used for the surveillance of adult mosquitoes: Centers for Disease Control and Prevention miniature light trap (CDC light trap) and BG-Sentinel trap (BioGents, Regensburg, Germany). However, despite the importance of the BG-Sentinel trap in surveillance programs in the United States, especially in the Southern states, its effectiveness in consistently and reliably collecting mosquitoes in rural and natural areas is still unknown. We hypothesized that BG-Sentinel and CDC light traps would be more attractive to specific mosquito species present in rural and natural areas. Therefore, our objective was to compare the relative abundance, species richness, and community composition of mosquitoes collected in natural and rural areas by BG-Sentinel and CDC light traps.

**Methods:**

Mosquitoes were collected from October 2020 to March 2021 using BG-Sentinel and CDC light traps baited with dry ice, totaling 105 trap-nights.

**Results:**

The BG-Sentinel traps collected 195,115 mosquitoes comprising 23 species from eight genera, and the CDC light traps collected 188,594 mosquitoes comprising 23 species from eight genera. The results from the permutational multivariate analysis of variance (PERMANOVA) and generalized estimating equation model for repeated measures indicate the BG-Sentinel and CDC light traps had similar sampling power.

**Conclusion:**

Even though BG-Sentinel traps had a slightly better performance, the difference was not statistically significant indicating that both traps are suitable to be used in mosquito surveillance in rural and natural areas.

**Graphical Abstract:**

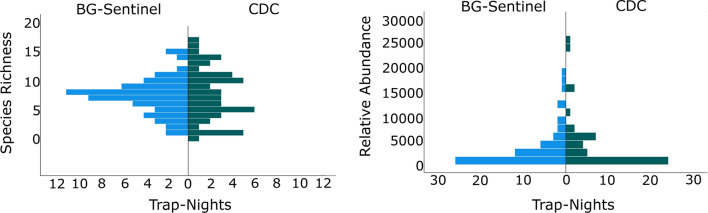

## Background

Vector-borne diseases are a major burden to public health. Currently, half of the world's population is at risk of vector-borne pathogen infections, resulting in approximately 1 billion infections every year [1–4]*.* In 2019, approximately 3 million cases of dengue were reported in the Americas [5], and vector-borne pathogen transmission is being reported more frequently not only in endemic areas [6–9], but also in non-endemic countries such as Croatia, France, and Italy [10–12].

The availability of effective drugs and vaccines is limited to a few pathogens and has reduced effectiveness in decreasing the prevalence and incidence of vector-borne pathogens [13–16]. In this context, controlling mosquitoes is widely accepted as the most effective way to prevent vector-borne pathogen transmission to humans and animals [17–19]. However, many mosquito vector species are responsible for transmitting different arboviruses and other pathogens, and the timely and precise detection of mosquito vector species in a given area is key for the development of targeted and effective control strategies [20].

Mosquito surveillance is a core component of integrated vector management, as surveillance programs are often the cornerstone for the development of mosquito control operations [18]. Most mosquito control districts in the United States operate surveillance systems to inform their control operations and guide control efforts including source reduction, chemical interventions, and environmental management [21–23]. However, consistently, accurately, and reliably assessing the presence and relative abundance of mosquito species is not a simple task often relying upon multiple trap types and approaches [23–26]. Furthermore, invasive mosquito species are an increasing threat to public health, and vector species such as *Aedes albopictus* and *Culex coronator* have expanded their range and abundance considerably in the last decade [27–32]. A reliable mosquito surveillance system should be able to early detect invasive species allowing stakeholders to implement control efforts to curb their proliferation and avoid their establishment.

Therefore, a surveillance system should be able to inform stakeholders regarding the relative abundance, species richness, and community composition of mosquitoes. However, different traps have different levels of attractiveness for different mosquito species, and choosing the right trap for collecting adult mosquitoes in different areas is key to achieving reliable and actionable results. In this context, two traps are the most commonly used for the surveillance of adult mosquitoes, Centers for Disease Control and Prevention miniature light trap (CDC light trap) and BG-Sentinel trap (BioGents, Regensburg, Germany). BG-Sentinel traps are the current gold standard for collecting *Aedes stegomyia* mosquitoes [33]. On the other hand, CDC light traps are considered more of a generalist trap that will attract a wider range of mosquito species, including *Anopheles* and *Culex* [23, 34, 35].

However, despite the importance of the BG-Sentinel trap in surveillance programs in the United States, especially in the Southern states, its effectiveness in consistently and reliably collecting mosquitoes in rural and natural areas in the United States is yet to be determined. We hypothesized that BG-Sentinel and CDC light traps have different levels of attractiveness and will be more attractive to specific mosquito species present in rural and natural areas leading to different outcomes of the community composition assessment. Therefore, our objective was to compare the relative abundance, species richness, and community composition of mosquitoes collected in natural and rural areas by BG-Sentinel and CDC light traps.

## Methods

### Collection of mosquitoes

**Fig. 1:**
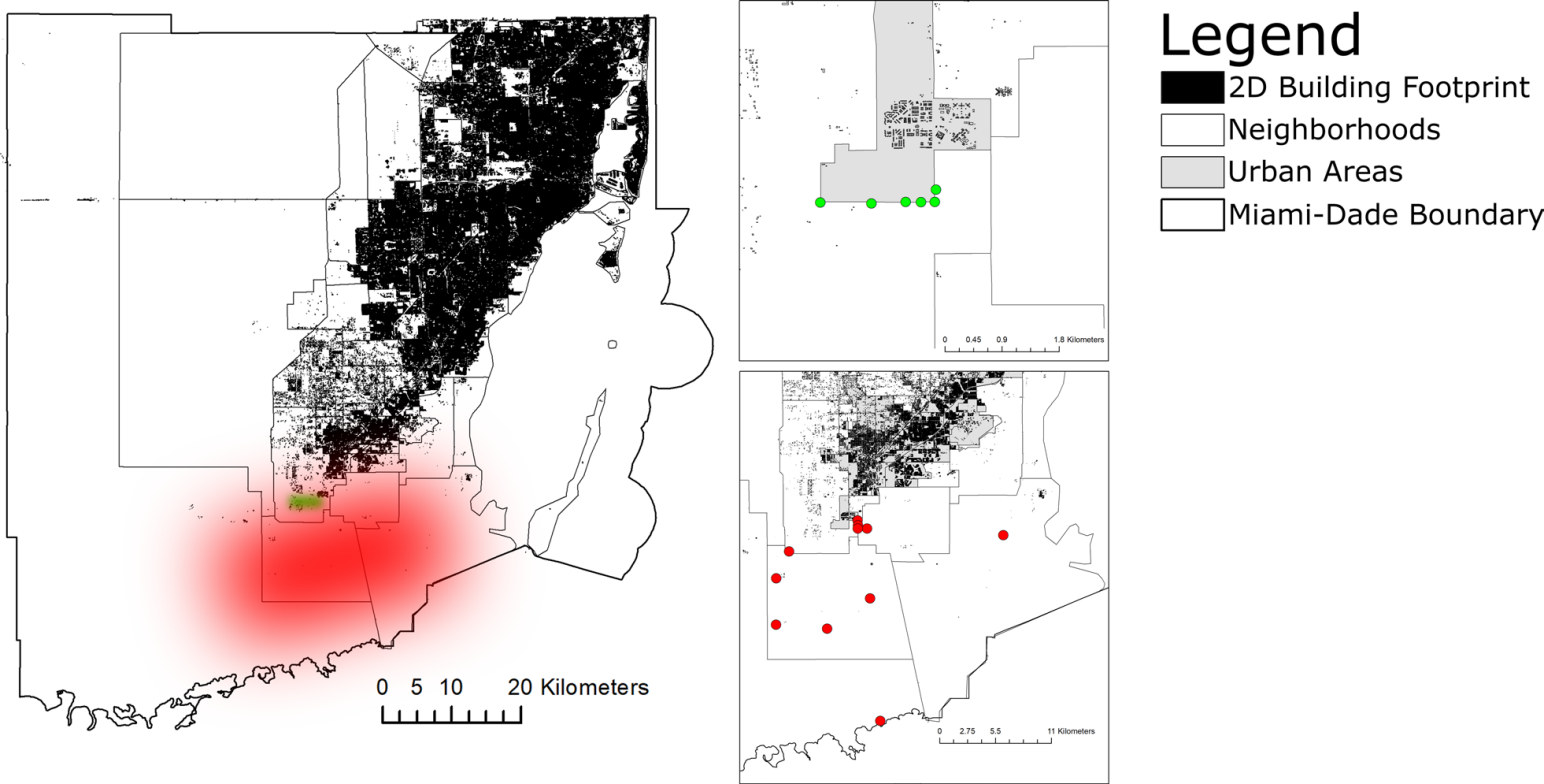
Map showing the location of the collection sites in Miami-Dade, Florida. The first set of 7 weeks of collection sites and trap locations are displayed in green, and the second set of experiments showing the 11 different collection sites in rural and natural areas and trap locations are displayed in red. The figure was produced using ArcGIS 10.2 (Esri, Redlands, CA) using freely available layers from the Miami-Dade County’s Open Data Hub—https://gis-mdc.opendata.arcgis.com/

Mosquitoes were collected from October 2020 to March 2021 using battery-powered BG-Sentinel 2 and CDC light traps, totaling 105 trap-nights. Firstly, we set three CDC and three BG-Sentinel traps at no more than 50 m from each other once a week for 24 h for 7 weeks in a rural area in the southern region of Miami-Dade County, Florida known for having great richness and abundance of mosquitoes (25°24′19.9″N; 80°30′03.9″W). Secondly, to test if the mosquito species richness and relative abundance after 7 weeks of collections would be similar in other areas of the Miami-Dade County, we selected 11 different collection sites in rural and natural areas, in which one BG-Sentinel and one CDC light trap were deployed together for 24 h at no more than 50 m from each other (Fig. 1). An insulated cooler with approximately 2 kg of dry ice was placed next to the traps as bait [36]. The traps were placed under similar environmental conditions hidden in the vegetation in shaded areas to protect the traps from the elements and enhance mosquito collections. CDC traps were set in tree branches at a height of approximately 1 m above ground level and BG-Sentinel traps were set directly on the ground. The collected mosquitoes were transported to the Miami-Dade County Mosquito Control Laboratory and subsequently morphologically identified to species using taxonomic keys [37].

### Statistical analyses

Biodiversity analyses were carried out for each trap type based on the Shannon, dominance, and equitability indices. The Shannon index takes into consideration species abundance and richness, therefore, less diversity results in lower values, and more diversity results in higher values [38]. Dominance (1-Simpson) index estimates the association between species richness and abundance, values close to 1 indicate the presence of dominant species whereas values closer to 0 imply a more even distribution between species richness and abundance [39]. The equitability index is calculated using the Shannon diversity index divided by the logarithm of the number of species [40]. This measures the evenness with which specimens are divided among the species in a given mosquito community. Analyses were carried out with 10,000 randomizations where each randomization is done without replacement using a 95% confidence interval (CI). To compare the mosquito species composition collected by CDC light traps and BG-Sentinel traps, we performed a permutational multivariate analysis of variance (PERMANOVA) with 9999 permutations based on Bray–Curtis distances [41, 42]. The data were organized into two groups (Group 1 = CDC light traps; and Group 2 = BG-Sentinel traps) to compare the mosquito species composition collected by the two different traps. Then, we used the SIMPER (similarity percentage) method to assess the contribution of each mosquito species to the observed differences between trap types [43]. Analyses were done using PAST v3.2 [44].

We performed a generalized estimating equation (GEE) model for repeated measures to assess differences in the species richness (number of species) and relative abundance of mosquitoes collected by BG-Sentinel and CDC light traps [45]. Species richness and relative abundance were used as dependent variables, trap type (BG-Sentinel and CDC light trap) as units, and collection date as repeated measures (longitudinal model). The model was done in SPSS v.28 software.

## Results

A total of 26 species from nine genera were collected by both BG-Sentinel and CDC light traps, totaling 383,709 specimens. The BG-Sentinel traps collected 195,115 mosquitoes comprising 23 species from eight genera. The CDC light traps collected 188,594 mosquitoes comprising 23 species from eight genera. *Aedes triseriatus*, *Coquillettidia perturbans*, and *Culex quinquefasciatus* were only collected by the BG-Sentinel traps, whereas *Aedeomyia squamipennis*, *Aedes condolescens*, and *Wyeomyia mitchelli* were only collected by the CDC light traps. The BG-Sentinel traps collected 6521 more mosquitoes than the CDC light traps. *Culex nigripalpus* was the most abundant species collected by both the BG-Sentinel (131,661) and CDC light traps (131,237), followed by *Culex erraticus* (BG-Sentinel = 16,127; CDC light trap = 13,442) and *Anopheles crucians* (BG-Sentinel = 15,515; CDC light trap = 13,878). *Aedes triseriatus* and *Ae. condolescens* were the least common species, being collected only once by a BG-Sentinel and a CDC light trap, respectively. *Mansonia dyari* was collected in larger numbers by BG-Sentinel traps (BG-Sentinel = 16,823; CDC light trap = 7984), on the other hand, *Culex panocossa* was collected in larger numbers by CDC light traps (BG-Sentinel = 2148; CDC light trap = 8387) (Table 1).

**Table 1:** Total number of mosquitoes collected by BG-Sentinel and CDC light traps in Miami-Dade County, Florida

Species	BG-Sentinel trap			CDC light trap			Grand total
	Males	Females	Total	Males	Females	Total	
*Aedeomyia squamipennis*				3	39	42	42
*Aedes albopictus*		17	17	8	3	11	28
*Aedes atlanticus*	10	5174	5184		2978	2978	8162
*Aedes condolescens*			0		1	1	1
*Aedes infirmatus*		27	27		55	55	82
*Aedes scapularis*		5	5		36	36	41
*Aedes taeniorhynchus*		55	55		101	101	156
*Aedes tortilis*		177	177		346	346	523
*Aedes triseriatus*		1	1				1
*Anopheles crucians*	192	15,323	15,515	32	13,846	13,878	29,393
*Anopheles quadrimaculatus*	2	3089	3091	3	4299	4302	7393
*Anopheles walkeri*		51	51		97	97	148
*Coquillettidia perturbans*		7	7				7
*Culex coronator*		124	124		64	64	188
*Culex erraticus*	27	16,100	16,127	77	13,365	13,442	29,569
*Culex interrogator*		206	206		379	379	585
*Culex nigripalpus*	62	131,599	131,661	176	131,061	131,237	262,898
*Culex panocossa*	10	2138	2148	117	8270	8387	10,535
*Culex quinquefasciatus*		5	5				5
*Mansonia dyari*		16,823	16,823		7984	7984	24,807
*Mansonia titillans*		3735	3735		5157	5157	8892
*Psorophora columbiae*		108	108		32	32	140
*Uranotaenia lowii*	2	24	26	8	43	51	77
*Uranotaenia sapphirina*	1	20	21	2	8	10	31
*Wyeomyia mitchellii*			0		3	3	3
*Wyeomyia vanduzeei*		1	1		1	1	2

The diversity indices yielded similar values for the mosquito community identified by both BG-Sentinel and CDC light traps. The mosquito community estimated by the BG-Sentinel traps yielded a dominance index of 0.47, whereas the CDC light traps yielded a dominance index of 0.5. The Shannon and equitability indices also yielded similar results for both traps 1.2 and 0.38, respectively (Table 2).

**Table 2:** Diversity indices values for the mosquito community identified by both BG-Sentinel and CDC light traps

Indices	BG-Sentinel traps	CDC light traps
Dominance	0.47 (CI: 0.4749–0.4799)	0.5 (CI: 0.4973–.5027)
Shannon	1.20 (CI: 1.198–1.209)	1.20 (CI: 1.195–1.207)
Equitability	0.38 (CI: 0.3821–0.3857)	0.38 (CI: 0.3811–0.3891)

Confidence interval (CI) = 95%

The PERMANOVA did not yield significant results for the comparison between the mosquito community comprising the mosquitoes collected by the BG-Sentinel and CDC light traps (F = 1.54; *P* = 0.11). The subsequent SIMPER analysis of the mosquito community showed that *Cx. nigripalpus*, *An. crucians*, and *Cx. erraticus* contributed the most to the observed differences (Table 3).

**Table 3:** SIMPER (similarity percentage) analysis of which species contributed the most to the observed differences comparing BG-sentinel and CDC light traps

Species	Average dissimilarity	Contribution (%)	Cumulative contribution (%)
*Culex nigripalpus*	44.36	54.98	54.98
*Anopheles crucians*	12.4	15.37	70.36
*Culex erraticus*	7.54	9.35	79.71
*Mansonia dyari*	6.18	7.66	87.38
*Anopheles quadrimaculatus*	3.34	4.15	91.53
*Culex panocossa*	2.40	2.97	94.51
*Mansonia titillans*	1.86	2.30	96.81
*Aedes atlanticus*	1.68	2.08	98.9
*Psorophora columbiae*	0.16	0.20	99.11
*Culex interrogator*	0.15	0.19	99.3
*Aedes tortilis*	0.15	0.19	99.49
*Aedes taeniorhynchus*	0.14	0.18	99.67
*Uranotaenia lowii*	0.06	0.07	99.75
*Anopheles walkeri*	0.05	0.06	99.81
*Culex coronator*	0.03	0.04	99.86
*Aedes infirmatus*	0.03	0.04	99.9
*Uranotaenia sapphirina*	0.03	0.03	99.94
*Aedes scapularis*	0.01	0.01	99.96
*Aedeomyia squamipennis*	0.01	0.01	99.97
*Wyeomyia mitchellii*	0.008	0.01	99.98
*Aedes albopictus*	0.008	0.01	99.99
*Coquillettidia perturbans*	0.002	0.002	100
*Wyeomyia vanduzeei*	0.001	0.001	100
*Culex quinquefasciatus*	0.0009	0.001	100
*Aedes triseriatus*	0.0004	0.0005	100
*Aedes condolescens*	0.0002	0.0003	100

**Fig. 2:**
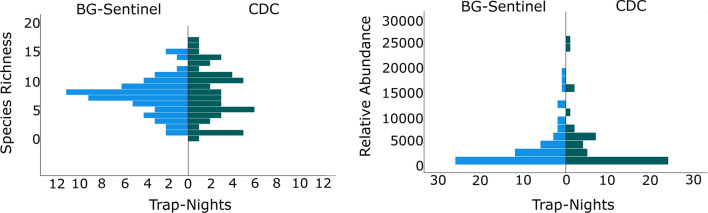
Comparison of the effectiveness of BG-Sentinel and CDC light traps in assessing species richness and relative abundance of mosquitoes in rural and natural areas of Miami-Dade, Florida

The results of the GEE models for repeated measures for species richness and relative abundance of mosquitoes collected by BG-Sentinel and CDC light traps showed no statistically significant differences between traps (Table 4). Even though the comparison of the species richness and relative abundance of the mosquitoes collected by the BG-Sentinel and CDC light traps were not significantly different, the BG-Sentinel traps collected more species per trap-night when compared to CDC light traps. Both the BG-Sentinel and CDC light traps had similar performances estimating the relative abundance of mosquitoes (Fig. 2).

**Table 4:** Results of the generalized estimating equation models for repeated measures for species richness and relative abundance of mosquitoes collected by BG-Sentinel and CDC light traps

Dependent variables	Parameters	Parameter estimates						Tests of model effects		
		Standard error	95% Wald CI		Wald Chi-square	*df*	*P*-value	Wald Chi-square	*df*	*P*-value
Species richness	Intercept	1.30	5.06	10.17	34.22	1	** > 0.001**	100.40	1	** > 0.001**
	Trap type	1.49	− 3.22	2.63	0.03	1	0.84	0.04	1	0.840
Relative abundance	Intercept	1616.40	844.55	7180.72	6.16	1	**0.013**	14.11	1	** > 0.001**
	Trap type	1995.68	− 4439.89	3383.01	0.07	1	0.79	0.07	1	0.790

## Discussion

BG-Sentinel and CDC light traps have been extensively used to successfully assess the relative abundance, species richness, and community composition of vector mosquitoes [23, 46, 47], and are essential for mosquito control operations. Our results show that the BG-Sentinel and the CDC light traps performed equally in assessing the species richness and relative abundance of mosquitoes. The number of mosquitoes collected by the BG-Sentinel and the CDC light traps varied less than 4%. Both the BG-Sentinel and the CDC light traps collected 23 species from eight genera from a total of 26 species from nine genera detected in total. The diversity indices yielded virtually identical results and the GEE model for repeated measures showed no significant differences between the performance of the BG-Sentinel and CDC light traps. However, even though the difference was not statistically significant, the BG-Sentinel traps had slightly superior performance; they collected more mosquitoes in total and yielded higher species richness in more trap-nights.

Most species collected by the BG-Sentinel and the CDC light traps during this study were fairly evenly distributed between traps. However, *Ma. dyari* and *Cx. panocossa* were the exceptions. Twice as many *Ma. dyari* were collected by BG-Sentinel traps, and *Cx. panocossa* was collected approximately 4 times more by CDC light traps. Furthermore, BG-Sentinel traps failed to collect *Ad. squamipennis*, and CDC light traps failed to collect *Cq. perturbans*. These results indicate that even though the performance in collecting mosquitoes of the BG-Sentinel and the CDC light traps were not statistically significantly different, some species were more attracted by one trap instead of the other.

The results of the PERMANOVA showed no significant differences in the mosquito community collected by the BG-Sentinel and the CDC light traps, in agreement with the GEE model for repeated measures and the diversity indices. Furthermore, the subsequent SIMPER analysis showed that the most abundant mosquitoes contributed the most to the observed differences indicating that the performance of the traps in collecting mosquitoes was similar. These results indicate that both trap types had similar performances in collecting rare species or failing to collect specific species.

Studies done in Europe and China had similar results to the ones obtained in this study, in which BG-Sentinel traps performed equally or were slightly superior to CDC light traps [48, 49]. However, in another study done in South Africa CDC traps had a superior performance in comparison to BG-Sentinel traps [50]. Local environmental and climatic conditions have a major influence on the development and proliferation of mosquitoes and greatly affect their behavior and ecology [51–54]. Therefore, locally assessing the effectiveness of the traps used to investigate the mosquito community composition, species richness and relative abundance in rural and natural areas is essential to improve the reliability and usefulness of mosquito surveillance and early warning systems.

Our results showed the presence of mosquito vector species in the rural and natural areas surveyed in this study. Among them two primary vectors of pathogens were collected in large numbers, *Anopheles quadrimaculatus* (primary vector of malaria in the Americas) and *Cx. nigripalpus* (primary vector of West Nile virus) [53, 55]. Mosquito surveillance in rural and natural areas bordering urban areas is key to avoiding vector-borne pathogen transmission to human and animal populations [56–58]. Anthropogenic alterations in the environment such as deforestation and defaunation often lead to habitat fragmentation [59]. Such human-made environmental alterations have a substantial impact on the mosquito community composition, relative abundance, and species richness. The behavior and ecology of mosquito vector species that are anthropophilic but non-synanthropic are greatly affected by anthropogenic alterations in the environment [60–63]. As a consequence, these mosquito vector species will invade urban areas seeking resources and will increase their contact with humans leading to a higher risk of pathogen spillover to human populations [64–66]. Therefore, the correct identification of the relative abundance and species richness in rural and natural areas bordering urban areas is key to determining the risk of vector-borne pathogens to humans [67–69].

Reliable and effective mosquito surveillance systems are key for the early detection of invasive species and to help to prevent their establishment as well as to inform mosquito control operations and guide control efforts [70–72]. Even though BG-Sentinel and the CDC light traps have had similar performances and were able to assess the community composition of mosquitoes in rural and natural areas, other sampling methods should also be considered to improve the effectiveness of surveillance systems. Immature mosquito surveillance systems should also be considered as the information obtained from such surveillance systems is complementary to adult mosquito surveillance systems, providing important information on what aquatic habitats are being used by each species and where the highest relative abundance levels of immature mosquitoes are concentrated [24]. Gravid traps are also an important tool since they use a different approach than traps that mimic a host (e.g., BG-Sentinel and the CDC light traps), and thus potentially complementing the sampling power of the surveillance system [73–75].

Mosquito collections were done between October 2020 and March 2021, and therefore, we were unable to assess all weather and season variations that would have provided further insight into the population dynamics and the mosquito community composition. CDC light traps and BG-Sentinel traps were not rotated in the first set of experiments due to the need to tie CDC light traps to tree branches. For this reason, the traps were set in the same environment at no more than 50 m from each other to avoid biases and inconsistencies in the collections.

## Conclusion

The results of the BG-Sentinel and CDC light traps in assessing the relative abundance and species richness of mosquitoes in rural and natural areas of Miami-Dade indicate that both traps performed equally, yielding similar results in all analyses. Therefore, we were able to reject the hypothesis that BG-Sentinel and CDC light traps would be more attractive to specific mosquito species present in rural and natural areas. Even though BG-Sentinel traps had a slightly better performance, the difference was not statistically significant indicating that both traps are suitable to be used in mosquito surveillance in rural and natural areas.

## Data Availability

All data generated or analyzed during this study are included in this published article.
